# The endogenous subcellular localisations of the long chain fatty acid-activating enzymes ACSL3 and ACSL4 in sarcoma and breast cancer cells

**DOI:** 10.1007/s11010-018-3332-x

**Published:** 2018-02-15

**Authors:** Yassmeen Radif, Haarith Ndiaye, Vasiliki Kalantzi, Ruth Jacobs, Andrew Hall, Shane Minogue, Mark G. Waugh

**Affiliations:** 1 Lipid & Membrane Biology Group, University College London, Floor U3, Royal Free Hospital Campus, Rowland Hill Street, London, NW3 2PF UK; 2 Sheila Sherlock Liver Centre, Royal Free London NHS Foundation Trust and UCL Institute for Liver and Digestive Health, University College London, London, UK

**Keywords:** Fatty acid, Lipid, Endosome, TGN, Endoplasmic reticulum, ACSL

## Abstract

Fatty acid uptake and metabolism are often dysregulated in cancer cells. Fatty acid activation is a critical step that allows these biomolecules to enter cellular metabolic pathways such as mitochondrial β-oxidation for ATP generation or the lipogenic routes that generate bioactive lipids such as the inositol phospholipids. Fatty acid activation by the addition of coenzyme A is catalysed by a family of enzymes called the acyl CoA synthetase ligases (ACSL). Furthermore, enhanced expression of particular ACSL isoforms, such as ACSL4, is a feature of some more aggressive cancers and may contribute to the oncogenic phenotype. This study focuses on ACSL3 and ACSL4, closely related structural homologues that preferentially activate palmitate and arachidonate fatty acids, respectively. In this study, immunohistochemical screening of multiple soft tissue tumour arrays revealed that ACSL3 and ACSL4 were highly, but differentially, expressed in a subset of leiomyosarcomas, fibrosarcomas and rhabdomyosarcomas, with consistent cytoplasmic and granular stainings of tumour cells. The intracellular localisations of endogenously expressed ACSL3 and ACSL4 were further investigated by detailed subcellular fractionation analyses of HT1080 fibrosarcoma and MCF-7 breast cancer cells. ACSL3 distribution closely overlapped with proteins involved in trafficking from the *trans*-Golgi network and endosomes. In contrast, the ACSL4 localisation pattern more closely followed that of calnexin which is an  endoplasmic reticulum resident chaperone. Confocal immunofluorescence imaging of MCF-7 cells confirmed the intracellular localisations of both enzymes. These observations reveal new information regarding the compartmentation of fatty acid metabolism in cancer cells.

**Electronic supplementary material:**

The online version of this article (10.1007/s11010-018-3332-x) contains supplementary material, which is available to authorized users.

## Introduction

Altered cellular energetics is a recently proposed hallmark of cancer [1]. In this context, upregulated lipid metabolism is one of the most striking metabolic changes observed in many tumour cells [2]. This metabolic reprogramming can involve an increased uptake of dietary-derived fatty acids from the circulation; increased de novo lipogenesis to provide membrane constituents and signalling lipids; and ATP generation through β-oxidation of fatty acids in the mitochondria [2]. Central to all these upregulated lipid-dependent events are the long chain fatty acyl CoA synthetase (ACSL) enzymes which activate fatty acids intracellularly in a reaction requiring Co-enzyme A and ATP to produce fatty acyl CoA [3, 4]. ACSL-dependent fatty acid activation is an essential prerequisite for entry of these lipid molecules into the metabolic pathways that sustain cell proliferation and movement. The ACSL enzymes preferentially, although not exclusively, activate subsets of fatty acids based on their particular acyl chain length and degree of saturation. In this project, we focus on ACSL3 and ACSL4, which are closely related structural homologues. Both of these enzymes can activate arachidonate—an ω-6 polyunsaturated fatty acid but they differ in their overall substrate specificities, for example, ACSL3 more effectively utilises saturated fatty acids such as palmitate, laurate and myristate [3].

ACSL3 and ACSL4 also differ in terms of their subcellular distributions and organelle associations. There are some reports revealing that ACSL4 is enriched at specialised sub-regions of the endoplasmic reticulum that forms tight physical contact with mitochondria [5, 6]. These sub-domains have been termed mitochondrial-associated membrane (MAM) domains and are important for intracellular lipid anabolism and Ca^2+^ homeostasis (reviewed in [7, 8]). However, an ACSL4 MAM localisation has not been found in every cell type, and there is strong evidence that this isoform can localise to other organelles including endosomes [9], the secretory pathway [10], peroxisomes [6] and at the plasma membrane [11]. The structural determinants underlying ACSL4 targeting to different membranes are not yet known, but it has been suggested that for the neuronal-specific splice variant, a putative N-terminal small hydrophobic region could function as integral transmembrane anchor [11]. For ACSL3, there is also some disagreement in the literature regarding its steady-state subcellular localisations. Several reports have demonstrated that in cells with a high capacity for lipid storage such as hepatocytes, a pool of ACSL3 can be detected at the endoplasmic reticulum and on lipid droplets [12–14]. Moreover, structural modelling and mutation studies indicate that an N-terminal hydrophobic, hairpin structure is required both for ACSL3 catalytic activity and its association with the cytoplasmic face of the endoplasmic reticulum [14]. There are, however, also reports that ACSL3 is required for post-Golgi trafficking to the plasma membrane [10] and that it is principally targeted to the *trans*-Golgi network (TGN) [15]—at least in some cell types. Therefore, in order to understand more completely how fatty acid metabolism is compartmentalised in cancer cells, it is first necessary to determine the organelle-association patterns for endogenously ACSL3 and ACSL4 in different cancer subtypes and cell lines.

In addition to their key gatekeeper roles in fatty acid metabolism in normal tissues, ACSL3 and ACSL4 expression levels are frequently altered in cancer, and this is sometimes associated with a more aggressive metastatic phenotype and a poor prognosis [16–18]. Upregulated ACSL3-dependent fatty acid metabolism, and in particular augmented β-oxidation of ACSL3-derived lipid products, has recently been identified as a key determinant underlying the oncogenicity of lung tumour cells with a mutant KRAS driver mutation [19, 20]. In other recent studies, ACSL4 has been identified in non-biased screens as an enzyme required for ferroptosis [20, 21]—a particular form of iron-dependent cell death that can be triggered by cell death-inducing phospholipid oxidation [22]. ACSL4 can supply arachidonyl-CoA and adrenic-CoA for incorporation into phosphatidylethanolamine and to a lesser degree phosphatidylinositol, and thus, these downstream ACSL4 product species can therefore be viewed as pro-ferroptotic lipid molecules [23]. Given the emerging importance of ACSL3 and ACSL4 in cancer, we describe here a series of subcellular fractionation experiments aimed at elucidating and comparing the subcellular distributions of these enzymes in cancer cells. We chose to work on the HT1080 cell line since we are interested in the subcellular organisation of phosphatidylinositol metabolism in these cells and in particular, the intracellular targeting of phosphatidylinositol 4-kinases. In light of recent demonstrations that ACSL4 may be involved in channelling specific pools of acyl chains to inositol phospholipids and given that ACSL3 could potentially also be important in this regard, we sought to establish the subcellular distributions of these fatty acid-activating enzymes relative to key enzymes involved in inositol phospholipid metabolism and signalling. In addition, very little is known about the endogenous intracellular localisations of these ACSL isoforms in non-hepatic cells and in cancer cells generally,  thus addressing this knowledge gap that was another motivation for undertaking this study. Given that ACSL4 has been found necessary for ferroptosis in breast cancer cells, we also report our findings regarding the subcellular targeting of endogenously expressed ACSL3 and ACSL4 in MCF7 breast cancer cells.

## Materials and methods

### Materials

The HT1080 cell line which was a gift from Prof Berditchevski, University of Birmingham, UK. MCF7 breast cancer cells were purchased from ECACC (Salisbury, UK). Anti-ACSL4 (#sc-365230) used for western blotting subcellular fractions from HT1080 cells and in imaging confocal immunofluorescence studies was purchased from Santa Cruz Ltd (Wembley, UK). Anti-ACSL4 antibodies used in western blot experiments for MCF-7 subcellular fractions were purchased from Invitrogen (#PA5-27137) (Paisley, UK) and GeneTex (#GTX100260) (Wembley, UK). Anti-Rab11 (#71-5300) was also purchased from Invitrogen (Paisley, UK). Anti-ACSL4 used in immunohistochemistry experiments was purchased from Proteintech Europe (Manchester, UK). Separate antibodies raised against either the C- or N-terminus of ACSL3 (#R3279-2 and #R3279-1, respectively) for use in western blots were obtained from Abiocode (Agoura Hills, California, USA). Anti-calnexin (#ADI-SPA-860) was from Enzo biosciences (Exeter, UK), Anti-GM130 (#2296), Anti-syntaxin 6 (#610636), anti-EEA1 (610457), anti-caveolin (#610059) and anti-prenylcysteine lyase (#612048) were from BD Biosciences (Oxford, UK). Anti-EGFR (#2232), anti-VDAC (#4866) and anti-mitofusin-1 (#14739) were obtained from Cell Signalling Technology, Europe B.V. (Leiden, The Netherlands) as were anti-Akt (#9272), anti-PLCγ (#2822), anti-mouse HRP-tagged secondary (#7076) and anti-rabbit HRP-tagged secondary (#7074) antibodies. Anti-TIP47 (#sc-390981) was bought from Santa Cruz Ltd (Wembley, UK). Anti-Sigma Receptor-1 (#HPA018002) and anti-TOMM20 (#HPA011562) were purchased from Atlas Antibodies (Bromma, Sweden). Anti-HMGCR (#ab174830) was obtained from Abcam (Cambridge, UK). Anti-α-1,2-mannosidase (#045K4799) was purchased Sigma-Aldrich (Poole, Dorset, UK). The anti-phosphatidylinositol 4-kinase type IIα monoclonal antibody was raised in house and previously described [24]. All multiple tissue arrays (# T242, T244 and SO809a) were obtained from US Biomax (Rockville, USA). Dulbecco’s Modified Eagle’s medium (DMEM), foetal bovine serum and penicillin/streptomycin were purchased from Invitrogen (Paisley, UK). Protease inhibitor cocktail tablets (Complete™, without EDTA) were from Roche Diagnostics (Burgess Hill, West Sussex, UK). Tween-20 detergent, skimmed milk powder, HRP-linked cholera toxin B subunit from Vibrio cholera and Bradford reagent were bought from Sigma-Aldrich (Poole, Dorset, UK). Sodium-dodecyl-sulphate (SDS), glycerol, bromophenol blue and dithiothreitol (DTT) were from VWR International (Lutterworth, UK). All other reagents were from Sigma-Aldrich (Poole, Dorset, UK). Pre-cast criterion PAGE gels and clarity-enhanced chemiluminescence reagents were purchased from Bio-Rad Laboratories Ltd (Watford, UK).

### Cell culture

HT1080 and MCF-7 cells were maintained in DMEM supplemented with 10% foetal calf serum, 50 i.u./ml penicillin, and 50 µg/ml streptomycin. Cells were cultured on 145 mm dishes at 37 °C in 10% CO_2_.

### Equilibrium sucrose density gradient fractionation of HT1080 and MCF-7 cells

For each experiment, two 145-mm dishes of confluent cell monolayers were used. Cell culture dishes were placed on ice and the medium aspirated. The cell monolayers were quickly washed twice with ice-cold PBS pH 7.4. The PBS was decanted and replaced with 1 ml of ice-cold cell homogenisation buffer consisting of 10 mM Tris-HCl pH 7.4, 0.25 M sucrose plus Complete™ EDTA-free protease inhibitors. The cell monolayers were scraped into homogenisation buffer, and the cell suspension transferred to a hand-held loose-fitting Dounce homogeniser where the cells were disrupted by 10 strokes. The homogenised cell suspension was then centrifuged at 1000×*g* for 3 min to pellet out the nuclei and unbroken cells. The resultant supernatant was termed the post-nuclear supernatant (PNS), and this was used in subsequent subcellular fractionation procedures.

The supernatant was adjusted to 2 ml, transferred into a 13.2-ml ultra-clear tube (Beckman Coulter UK Ltd, High Wycombe UK) and layered on top of a 15–50% w/v sucrose gradient that was composed of 2 ml steps of 50%, 40% and 35%, and 1 ml of each of 30, 25, 20 and 15% w/v sucrose solutions dissolved in Tris 10 mM, EDTA 1 mM, EGTA 1 mM. Each layer of sucrose solution was added drop by drop down the side of the tube, following the meniscus as it rose, to prevent mixing of the layers. The tube was filled to within a few millimetres of the rim. Next, the tube was carefully placed in an SW41 Beckman swing-out rotor and centrifuged overnight at 150,000×*g* at 4 °C. Following centrifugation, the gradient fractions were collected, using a 1-ml pipette to carefully decant 12 × 1 ml fractions starting at the top of the sucrose gradient.

### Isolation of mitochondrial-associated membranes from MCF-7 cell

Mitochondrial-associated membranes were isolated from MCF-7 cells using a previously published method [25] with some minor adjustments. A PNS was prepared from 4 to 6 confluent 145 mm dishes of MCF-7 cells using the same buffer and methods as described for equilibrium sucrose density gradient fractionation. The PNS was then centrifuged at 10,200×*g* for 20 min at 4 °C to produce a supernatant consisting of cytosol and microsomes, as well as a crude mitochondrial pellet containing mitochondria along with intact MAM. The supernatant from this step was collected and placed on ice to be used at a later stage. The crude mitochondrial pellet was collected and suspended in 500 µl of buffer 1(10 mM Tris, pH 7.4, 1 mM EDTA, 1 mM EGTA, 250 mM mannitol).

MAM were subsequently separated from mitochondria using a Percoll density gradient. Percoll buffer (10 mM Tris, pH 7.4, 1 mM EDTA 1 mM EGTA, 225 mM mannitol, 30% v/v/ Percoll) was added to fill 2/3 of a 13.2 ml polycarbonate ultracentrifuge centrifuge tube. The crude mitochondrial suspension was layered carefully on top of the Percoll buffer, so that there was a visible line between the two layers. The remainder of the tube was filled to within a few millimetres of the rim with buffer 1 and was centrifuged at 95,000×*g* for 30 min at 4 °C, in a swinging bucket rotor (SW41TI Beckman Coulter OptimaTM LE80-K Ultracentrifuge) with deceleration set to zero. Following centrifugation the MAM fraction was visible as a thin, white layer above the mitochondria and was collected. The denser mitochondria were recoverable from around three quarters of the way down the tube, and were also collected. The MAM was diluted five-fold  in buffer 2 (10 mM Tris, pH 7.4, 1 mM EDTA, 1 mM EGTA, 225 mM mannitol) and centrifuged at 6200×*g* for 10 min at 4 °C. The resulting supernatant was collected and the pellet discarded.

The MAM and the supernatant containing microsomes/cytosol were each placed in a 12 ml polycarbonate centrifuge tube, with the remainder filled with buffer 1. The tubes were centrifuged at 100,000×*g* for 1 h at 4 °C. Following this, the MAM was visible as loose, floating white material at the bottom of the tube. The supernatant was removed, and the MAM was collected and suspended in 200 µl of homogenisation buffer. The microsomal pellet was also collected and suspended in 200 µl of homogenisation buffer, and the cytosolic supernatant was discarded.

The mitochondria fraction from the Percoll gradient step was diluted five-fold in buffer 1, and then washed three times by centrifugation at 10,400×*g* for 10 min each. After each spin, 500 µl of the supernatant was discarded, and replaced by buffer 1. After the third spin, all of the supernatant was discarded, and the mitochondrial pellet was collected and suspended in 200 µl of buffer 2.

### Lipid-raft detection with cholera toxin b subunit

A dot-blot method previously described by us was used to detect lipid-raft-enriched membrane fractions [26]. Equal volume 1 µl samples from each subcellular fraction were dotted onto nitrocellulose membrane and allowed to dry at room temperature before being probed with probed with horse radish peroxidase (HRP)-conjugated cholera toxin B subunit (1:20,000). Bound HRP-conjugated cholera toxin B subunit was detected using Clarity enhanced chemiluminescence reagents and the FluorChem M gel imaging system (from ProteinSimple, Oxford, UK).

### Western blotting of HT1080 subcellular fractions

Aliquots from each subcellular fraction were mixed with an equal volume of 2 × SDS-PAGE sample buffer containing DTT and heated for 10 min at 80 °C on a heat block. Samples were cooled to room temperature and used immediately or stored at − 20 °C. The protein content of each fraction was separated by polyacrylamide gel electrophoresis on 12% pre-cast Criterion gels (Bio-Rad Laboratories Ltd, Watford, UK). Equal volume samples, typically 30 µl from each sucrose gradient fraction were analysed in each experiment. The separated proteins were transferred to PVDF membranes using the iBlot system (Thermo Fisher Scientific, Life Technologies Ltd, Paisley, UK) and blocked for 1 h at room temperature in Tris 5 mM, NaCl 137 mM, 0.1% Tween-20 pH 7.4 buffer (TBST) containing 5% w/v skimmed milk powder. Antibodies were added overnight at 4 °C and following washing in TBST the addition of secondary HRP-conjugated antibodies, blots were visualised using the Clarity enhanced chemiluminescence reagent and the FluorChem M gel imaging system (ProteinSimple, Oxford, UK) or in some cases by exposure to X-ray film.

### Image and statistical analyses

Image J software (https://imagej.net/ImageJ) was used to quantify signal intensity from both western and dot blots. Data were analysed and plotted using GraphPad Prism 5 software (GraphPad, San Diego, USA). For the cholera toxin B subunit dot-blots, the means ± SEM of triplicate determinations were calculated using GraphPad Prism 5 software.

### Immunohistochemistry of sarcoma multiple tissue arrays

Protein expression of ACSL3 and ACSL4 in tissue microarray sections of tumour samples was demonstrated using rabbit polyclonal primary antibodies raised against ACSL3 [#R2379-1 from Abiocode (Agoura Hills, California, USA)] and ACSL4 [#22401-1-AP from Proteintech Europe (Manchester, UK)], respectively. Antibody binding was detected and visualised with the Novolink TM max polymer detection system kit (Novocastra, Leica Microsystems (UK) Ltd, Milton Keynes, UK). Sections were dewaxed in xylene and taken to water through graded industrial denatured alcohol. Antigen retrieval was achieved by microwaving at 700 W for 15 min in 1 L of pH 6.0 sodium citrate buffer. The slides were then soaked in wash buffer, TBS with 0.04% Tween-20 for 5 min, blocked in the peroxidase blocking solution for 5 min then washed in wash buffer for 5 min. The slides were blocked for non-specific binding of the post primary using the kit’s protein block. The primary antibody was diluted to 1:300 for ACSL3 and 1:250 for ACSL4 and the slides were incubated for 1 h at room temperature then rinsed in TBS wash buffer. The slides were then placed for 25 min in the post-primary solution, 25 min in the polymer solution and developed with 3,3′ di-amino-benzidine with a 5 min buffer wash between each of the steps. Slides were counterstained with Mayer’s Haematoxylin for 3 min. All sections were dehydrated in graded IDA, cleared in xylene and were mounted with DPX (Leica Microsystems (UK) Ltd, Milton Keynes, UK). The slides were observed using a Zeiss Axioskop 40 (Zeiss, Cambridge, UK) and images were captured with an Axiocam IcC5 using Zeiss Axiovision (version 4.8.2).

### Confocal laser-scanning immunofluorescence microscopy

Prior to staining, cells were fixed and permeabilised by treatment with − 25 °C methanol for 2 min, then immunostained with various antibodies. When staining with anti-ACSL3 immunostaining experiments cells were fixed in formalin and detergent permeabilised, as described [27]. Cell nuclei were counterstained with Hoechst 33342 (Thermo Fisher Scientific, Life Technologies Ltd, Paisley, UK) and following this the coverslips were mounted in ProLongGold anti-fade reagent (Thermo Fisher Scientific, Life Technologies Ltd, Paisley, UK). Cells were imaged as described previously [27] using a Zeiss LSM 510 Meta laser-scanning confocal microscope system.

## Results and discussion

### Profiling ACSL3 and ACSL4 expression in soft-tissue tumour microarrays

**Fig. 1:**
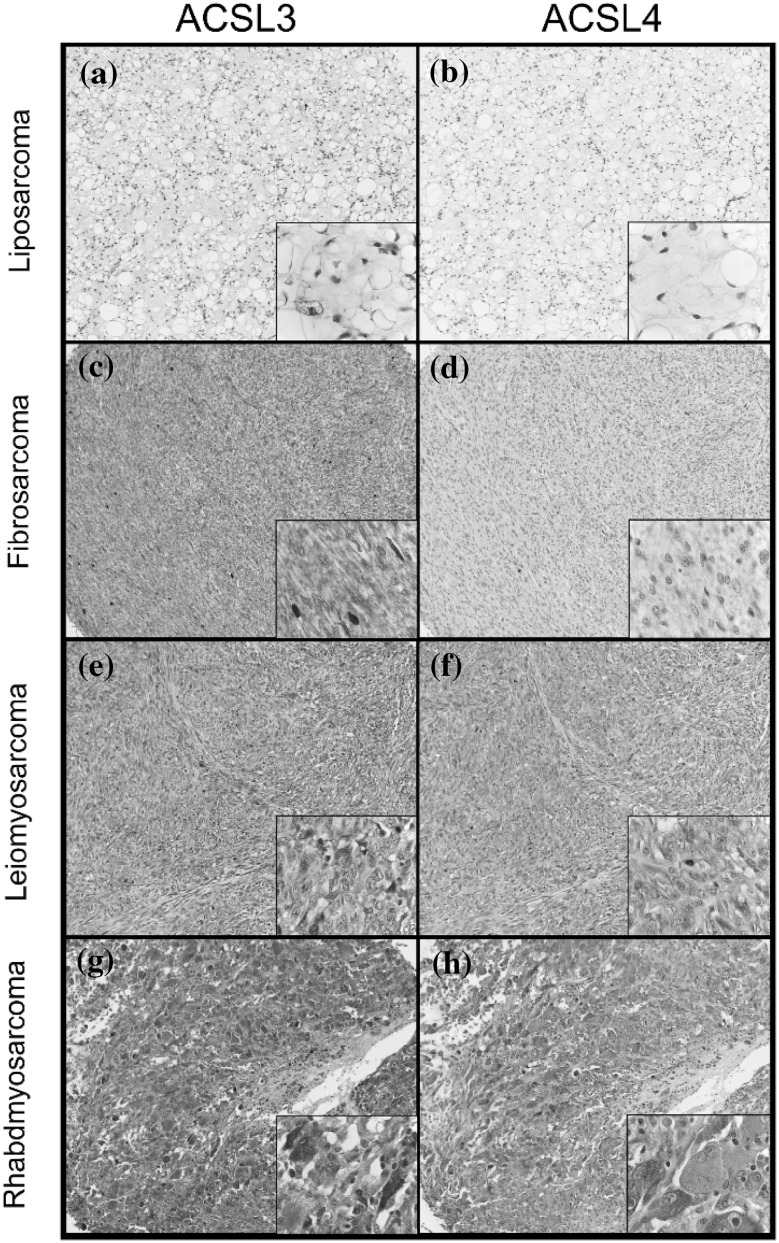
Immunohistochemistry reveals differential expression of ACSL3 and ACSL4 in sarcomas. Multiple tissues arrays were probed using antibodies specific for either ACSL3 or ACSL4. Representative images are shown for ACSL3 and ACSL4 immunohistochemical staining of matched patient samples from **a** and **b** liposarcoma, **c** and **d** fibrosarcoma, **e** and **f** leiomyosarcoma, and **g** and **h** rhabdomyosarcoma cores on multiple tissue arrays. Examples of negative and weak staining are presented in panels **a, b** and **d**, and high-intensity staining in the remaining panels The main image in each panel ×10 objective magnification, and each inset is ×100 objective magnification. Cytosplamic staining can be seen in higher-magnification inset images in panels **c**, and **e**–**h**. Note that, while ACSL3 staining was classified as weak overall in liposarcomas, it was nevertheless possible to visualise ACSL3 staining on the perimeter of lipid droplets in these samples **a** inset

As we were interested in elucidating the subcellular localisations of ACSL3 and ACSL4 in a fibrosarcoma cell line and since the expression levels of both enzymes are known to be altered in different cancers, we decided to investigate if the expression levels of these proteins were altered in soft-tissue tumours. To this end, we used isoform-specific antibodies and immunohistochemistry to probe a soft-tissue tumour microarray containing cores from 80 different clinical cases (Fig. 1). Using this approach, we found high or moderate-intensity staining for ACSL3 in 4/8 fibrosarcoma samples on the array. Moderate- or high-intensity signals were also frequently observed with leiomyosarcoma (10/20 samples) and some rhabdomyosarcomas (5/20 samples), but there was a more mixed pattern of ACSL3 expression in dermatofibrosarcomas (Table S1). However, there was consistently weak staining for ACSL3 in almost all liposarcoma cores (18/20 samples) (Fig. 1). Compared to ACSL3, moderate- or high-intensity staining for ACSL4 was less frequently observed across all soft-tissue tumour types examined (Supplementary Table 1). Notable exceptions to this trend were leiomyosarcomas where high or moderate ACSL4 staining was detected in (8/20 samples) and rhabdomyosarcomas (5/20 samples). It is important to note that we could not find any evidence for coincident regulation of ACSL3 and ACSL4 expression levels in these patient samples. In further experiments using smaller tissue microarrays for ACSL3 and ACSL4, we were able to readily detect staining for ACSL4 specifically in fibrosarcoma sections (4/4), but not in other soft-tissue tumours or normal tissue (Table 2S). These results indicated that ACSL4 expression was upregulated in some fibrosarcomas. For ACSL3, we again found consistently high-intensity signals for fibrosarcoma samples (4/4) in a third tissue microarray (Table S3). Under higher 100x magnification, both enzymes exhibited an intracellular, granular and cytoplasmic staining pattern in malignant cells (Fig. 1). In addition, antibodies directed against ACSL3, but not ACSL4, clearly decorated the perimeter of lipid droplets in liposarcoma samples (Fig. 1a), and this result aligns with the previously reported lipid droplet localisation for ACSL3 in adipogenic cells [13, 14]. In summary, these results confirmed that both enzymes were expressed in a subset of soft-tissue tumours and also that they were localised to intracellular membranes in tumour cells.

### Equilibrium density gradient fractionation of HT1080 cells

To gain further insights into the intracellular localisations of ACSL3 and ACSL4, we performed subcellular fractionation experiments using equilibrium sucrose density gradients. Post-nuclear supernatants were prepared from HT1080 fibrosarcoma cells on an equilibrium 15–50 w/v % sucrose density gradient. This technique was used as it separates out organelles according to their equilibrium-buoyant densities which are characteristic physical properties determined by the intrinsic protein and lipid compositions of each membrane compartment. Following overnight ultracentrifugation, 12 × 1 ml subcellular fractions were collected; fractions 1 and 2 represent the region of the gradient where the post-nuclear supernatant was loaded, and fractions 3–12 correspond to sucrose concentrations increasing from 15 to 50% w/v.

**Fig. 2:**
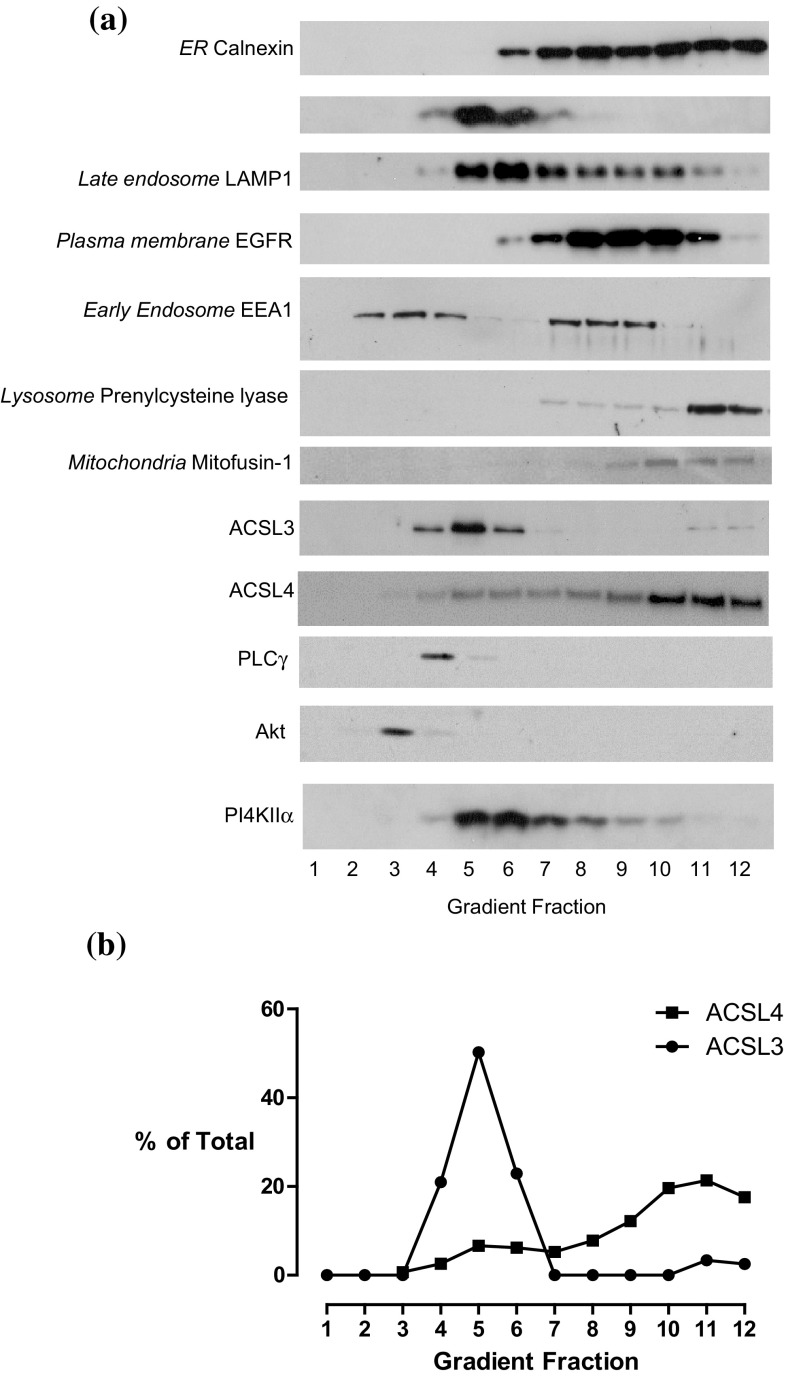
Equilibrium distributions of organelle marker proteins, ACSL3 and ACSL4 in HT1080 sucrose density gradient fractions. **a** Equal volumes of all HT1080 subcellular fractions were subjected to SDS-PAGE separation and immunoblotted for the ER marker protein calnexin; the TGN-endosomal protein syntaxin-6; the late endosomal protein LAMP1; the plasma membrane-associated EGFR, the early endosome-recruited protein EEA1; the lysosomal protein prenylcysteine lyase and the mitochondrial protein mitofusin-1, ACSL3 and ACSL4, and the inositol phospholipid-dependent enzymes Akt, PLCγ and PI4KIIα. Data presented are representative of experiments repeated 3–4 times with similar results. **b** The relative normalised distributions of anti-ACSL3 and anti-ACSL4 immunoreactivities in the gradient fractions. Western blotting signals were quantified using imageJ software. Data are representitive of experiments repeated 3–4 times with similar results

The distributions of different organelles were assessed by western blotting equal volume samples from each density gradient fraction with antibodies raised against proteins known to be enriched at particular subcellular compartments (Fig. 2). Using this approach, we found that immunoreactivity for the endoplasmic reticulum chaperone protein calnexin—a protein which reportedly distributes between MAM and non-MAM endoplasmic reticulum domains [28], gradually increased from fractions 6 to 12, with the strongest signals apparent in dense fractions 11 and 12. Immunoreactivity for the mitochondrial outer membrane protein mitofusin-1 [29, 30] was found between fractions 8–12 and peaked in fraction 10. LAMP1 a protein which trafficks from the TGN to lysosomes and which is particularly enriched at late endosomes [31], and also the TGN/endosomal protein syntaxin-6 [32] both peaked in fractions 5 and 6. Blots were also carried out to detect the presence of the early endosomal marker protein EEA1, and this protein was found in gradient fractions 2–4 and 7–9 corresponding to the cytosolic- and membrane-bound pools of this protein, respectively. The position of the plasma membrane in the gradient was ascertained by blotting for the epidermal growth factor receptor (EGFR), and we found that the main signal for this protein was detected in fractions 7–11 but was most pronounced in fractions 8–10. This immunoreactivity distribution pattern can be rationalised on the basis of the EGFR localising to biophysically distinct domains of the plasma membrane [33] and also partly to endosomal fractions [27]. Finally, the location of lysosomes in the gradient was determined by blotting for the lysosomal membrane protein prenylcysteine lyase [34], and the signal for this enzyme was confined to the densest fractions 11 and 12. In summary, the results of this western blotting characterisation confirmed that the gradient effectively separated out different organelles on the basis of their intrinsic equilibrium-buoyant densities.

Having established that different organelles and membrane compartments had characteristic density separation profiles we next sought to determine the subcellular distributions of endogenously expressed ACSL3 and ACSL4 by western blotting with isoform-specific antibodies (Fig. 2). Using this approach, we observed that ACSL3 was predominately found in the middle of the gradient between fractions 4 and 6 with a peak signal detected in fraction 5 thus mirroring the distributions of the TGN-endosomal protein syntaxin-6. In addition, a minor pool of ACSL3 was detected in the dense fractions 11 and 12 which closely overlapped with immunoreactivity for the lysosomal protein prenylcysteine lyase. This gradient localisation profile suggested that ACSL3 is a protein enriched on TGN/endosomal membranes with a small amount of the protein being trafficked to lysosomes. By way of contrast, immunoreactivity for the closely related homologue ACSL4 gradually increased in intensity in the dense region of the gradient beginning with fraction 5 but reaching a broad maximum between fractions 10 and 12. This pattern of gradient distribution closely paralleled that of the endoplasmic reticulum protein calnexin and strongly indicated that ACSL4 likewise distributed throughout this membrane network. Most strikingly, when we plotted the normalised gradient distribution profiles of ACSL3 and ACSL4 we found that they were very different which further strengthened the idea that these proteins have different subcellular localisations with ACSL3 targeted to the TGN–lysosomal pathway and ACSL4 to the endoplasmic reticulum (Fig. 1b).

There is a possibility that ACSL3 could supply the arachidonate tails required for the signalling pool of stearoyl arachidonyl-containing inositol phospholipids [3]. To investigate if ACSL3 at the TGN co-distributed with proteins required for receptor-stimulated inositol phospholipid signalling, we blotted for the presence of Akt and PLCγ. Using this approach, we observed the peak immunoreactivities for both of these enzymes were in the buoyant region of the gradient and were well separated from ACSL3 (Fig. 2). By way of contrast, we found that the peak immunoreactivity for phosphatidylinositol 4-kinase type IIα (PI4KIIα) did co-fractionate with ACSL3 in fractions 5 and 6 of the gradient. PI4KIIα is an enzyme which previous studies have found as being localised to both endosomes [35] and the TGN [36] and which generates an intracellular pool of phosphatidylinositol 4-phosphate that functions in vesicular trafficking. These results are consistent with the main cellular pool of ACSL3 localising to membranes involved in intracellular TGN-endosomal trafficking.

**Fig. 3:**
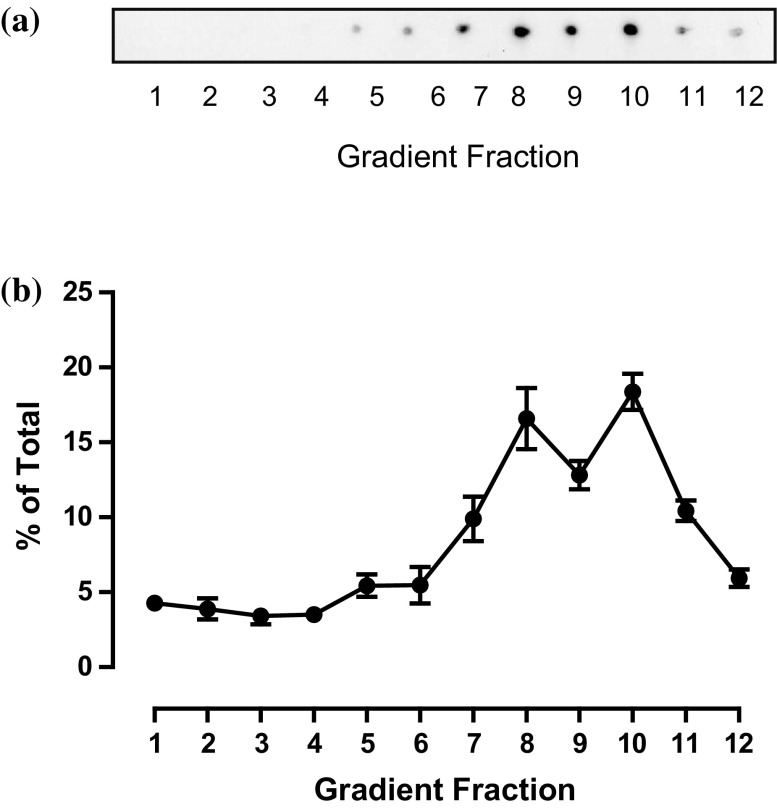
Dot blot showing the gradient distribution of glycosphingolipid-enriched lipid-raft fractions. **a** Equal volumes of all HT1080 subcellular fractions were dotted on to nitrocellulose membranes and probed with HRP-linked cholera toxin B subunit. **b** The distribution of the Cholera toxin B binding in the sucrose density gradient. Dot blotting signals were quantified using imageJ software. Data are presented as mean ± SEM of triplicate determinations

Whereas most of the pre-existing literature on ACSL3 localisation has reported an endoplasmic reticulum and/or lipid droplet localisations there is one report that this enzyme is targeted to lipid rafts at the TGN. Lipid rafts are cholesterol and sphingolipid-enriched microdomains of biological membranes implicated in the intramembrane organisation of signalling and trafficking pathways. To investigate if there was any overlap between ACSL3 and lipid-raft-enriched membranes, we probed for the presence of these structures using cholera toxin B subunit which is known to bind gangliosides such as GM1 and to report the presence of lipid rafts (reviewed in [37]). Using this approach, we found that the main cholera-toxin B reactive fractions were located in the dense region of the gradient and not in the main ACSL3-enriched membrane fractions (Fig. 3). This indicated that ACSL3 was unlikely to be targeted to the TGN-endosomal membranes solely through preferential association with lipid-raft membrane domains.

**Fig. 4:**
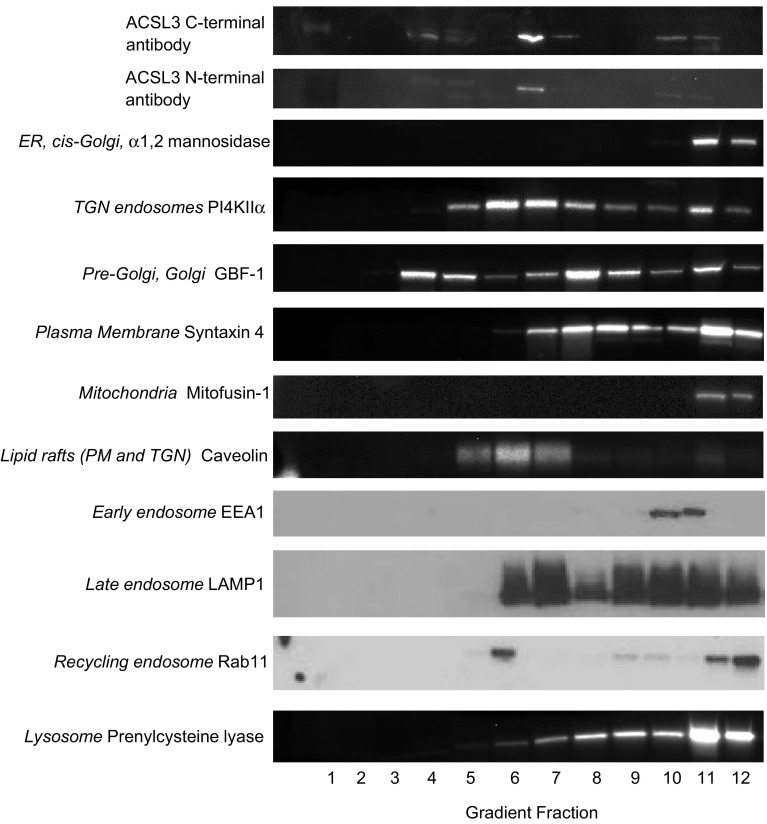
Equilibrium distributions of organelle marker proteins and ACSL3 in MCF-7 subcellular fractions. Equal volume samples from each MCF-7 sucrose gradient fraction were subjected to SDS-PAGE separation and immunoblotted for ACSL3 using two different antibodies, one raised against a C-terminal epitope and the other against an N-terminal epitope of the enzyme. The gradient distributions of a panel of organelle marker proteins was similarly determined by immunoblotting. Data presented are representitive of experiments repeated 2–3 times with similar results

To investigate if the localisations of both ACSL enzymes was solely a feature of HT1080 cells or a more general characteristic of cultured cancer cell lines, we extended our work and carried out similar subcellular fractionation and co-distribution studies with the MCF7 breast cancer cell line. In fractionated MCF7 cells, we observed that the endogenous distributions of ACSL3 and ACSL4 closely mirrored our findings with HT1080 cells. Using two different antibodies raised against either the C- or N-terminus of ACSL3, we found that this enzyme exhibited a clear peak of immunoreactivity in fraction 6 with a minor pool detected in the high density region of the gradient (Fig. 4). Again mirroring our findings with the fibrosarcoma cells, we observed that the main peak of ACSL3 immunoreactivity overlapped with TGN/endosome-targeted proteins such as PI4KIIα, caveolin and LAMP1, and had a different distribution profile and minimal overlap with markers for other compartments such as the plasma membrane localised protein syntaxin-4, the *cis*-Golgi and ER protein α−1, 2-mannosidase and the mitochondrial protein mitofusin-1. The minor ACSL3 pool in fractions 10 and 11 was found to co-fractionate with the endosomal marker EEA1 in this dense region of the gradient. These results demonstrate that ACSL3 has very similar intracellular membrane distribution patterns in both MCF-7 and HT1080 cells.

**Fig. 5:**
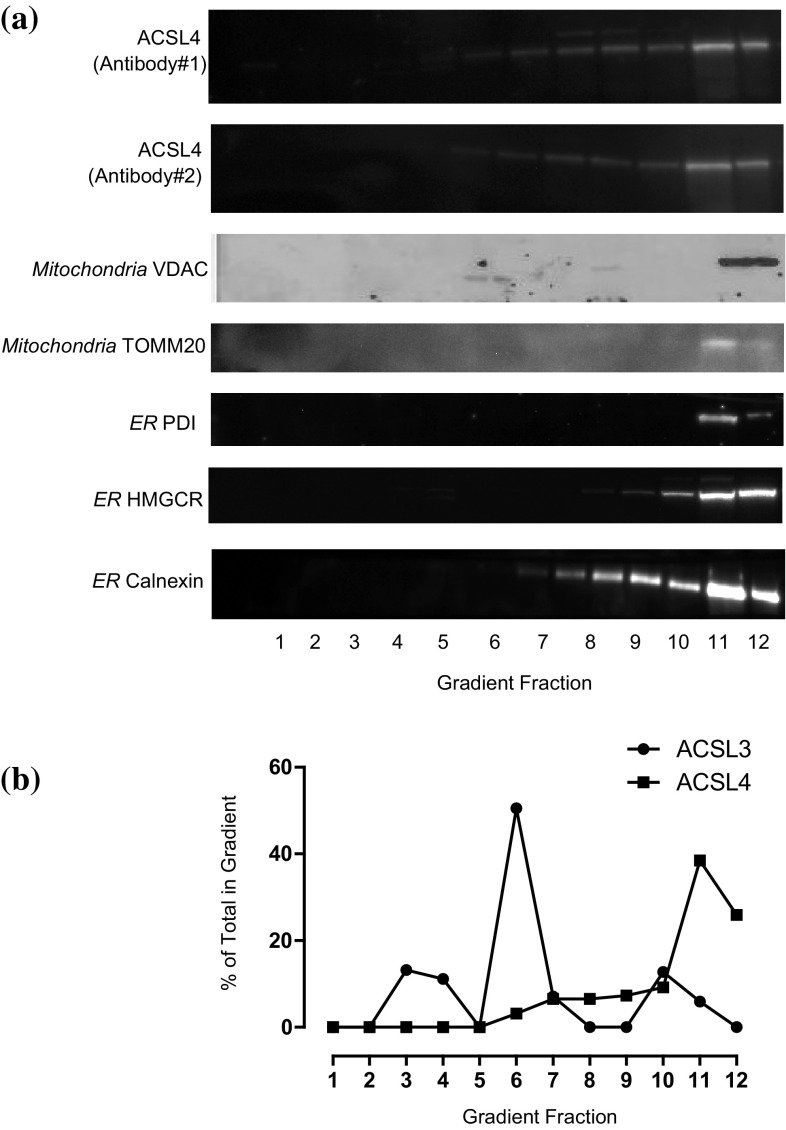
Equilibrium distributions of organelle marker proteins and ACSL4 in MCF-7 density gradient fractions. **a** Equal volume samples from each MCF-7 sucrose gradient fraction were subjected to SDS-PAGE separation and immunoblotted for ACSL4 using two different commercially available antibodies; antibody#1 was supplied by GeneTex and antibody#2 from Invitrogen. The gradient distributions of a panel of organelle marker proteins was similarly determined by immunoblotting. Data presented are representitive of experiments repeated 2–3 times with similar results. **b** The normalised distributions of anti-ACSL3 and anti-ACSL4 immunoreactivties in the gradient fractions. Western blotting signals were quantified using imageJ software. Data are representitive of experiments repeated 3–4 times with similar results

**Fig. 6:**
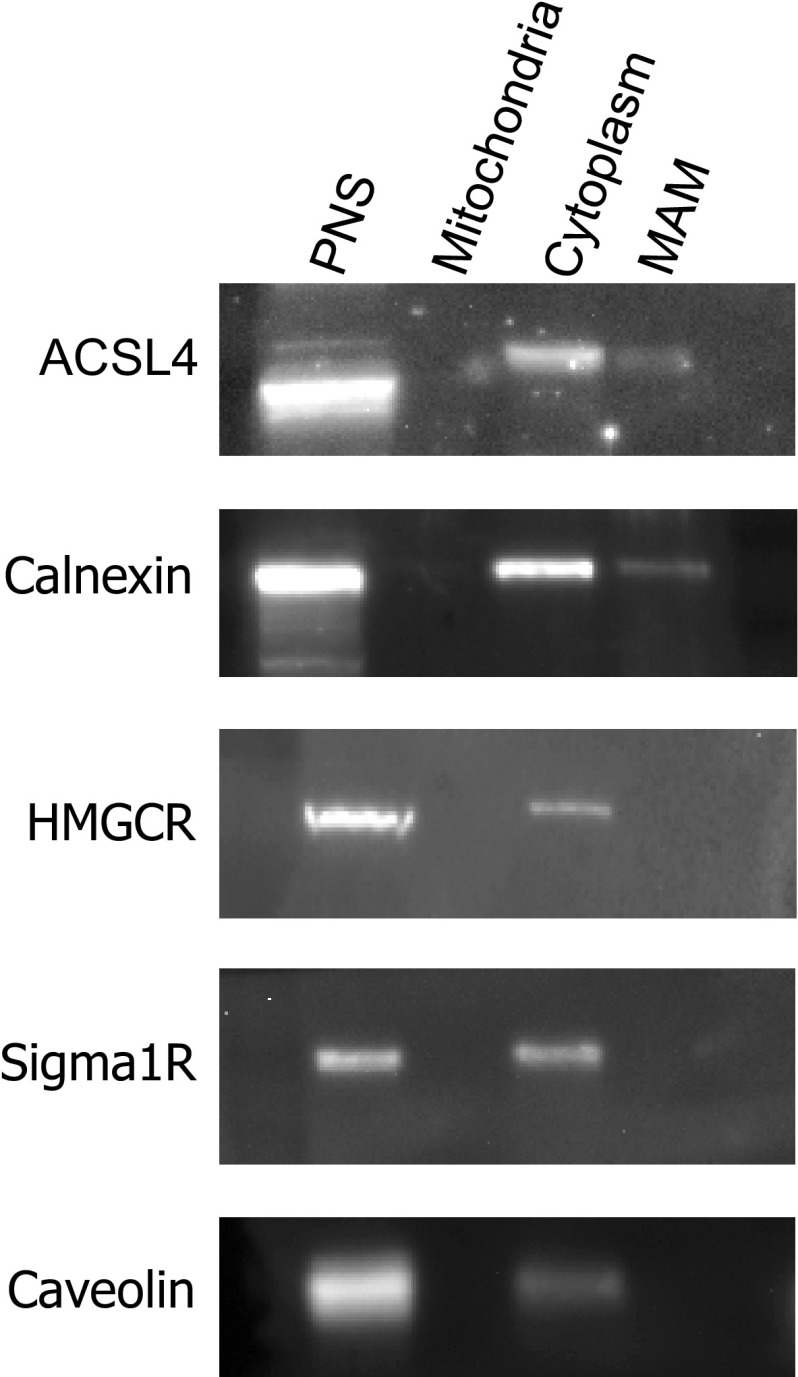
ACSL4 partially localises to MAM. MAM, cytoplasmic and mitochondrial fractions, were prepared from MCF-7 cells. Equal volumes of samples from each MCF-7 fraction were subjected to SDS-PAGE separation and immunoblotted for ACSL4, calnexin, caveolin, HMGCR and Sigma1R. Each panel represents the results of an experiment repeated twice with similar results

Using two different antibodies raised against ACSL4, we observed that ACSL4 had a markedly different distribution profile to ACSL3 in MCF-7 cell fractions. The distribution of ACSL4 very closely correlated with the endoplasmic reticulum proteins calnexin and HMG CoA reductase (HMGCR), and partially with PDI and Sigma1R—a protein proposed to localise specifically to ER MAM domains (Fig. 5). The dense region of the gradient corresponding to fractions 11 and 12 also contained all the immunoreactivity for the mitochondrial markers VDAC and mitofusin-1 and this could indicate that a proportion of the total cellular ACSL4 was associated with mitochondria in addition to MAM domains of the ER. To distinguish between these possible subcellular locations, we employed a well-established method to separate MAM from ER and mitochondria [25]. Using this experimental strategy, we were able to reproducibly isolate a membrane fraction with the expected visible and physical properties of MAM (Fig. 6), and western blotting confirmed that this membrane fraction was well separated from immunoreactivity for caveolin, HMGCR and unexpectedly, Sigma1R. This was not anticipated since Sigma1R has been proposed as a MAM marker protein in non-hepatic cells [25]. Furthermore, only a minor fraction of ACSL4 was present in the isolated MAM fraction with the bulk of the cellular compliment found in the cytoplasmic fraction. In this MAM fractionation scheme, as with the equilibrium sucrose density gradients, the distribution of ACSL4 closely mirrored that of calnexin—a protein known to distribute between MAM and non-MAM domains of the ER [38]. These findings prompted us to conclude that in a similar way to calnexin, ACSL4 was not restricted to MAM. Moreover, our results do not support the idea that either ACSL4 or Sigma1R are useful MAM marker proteins in all cell types.

**Fig. 7:**
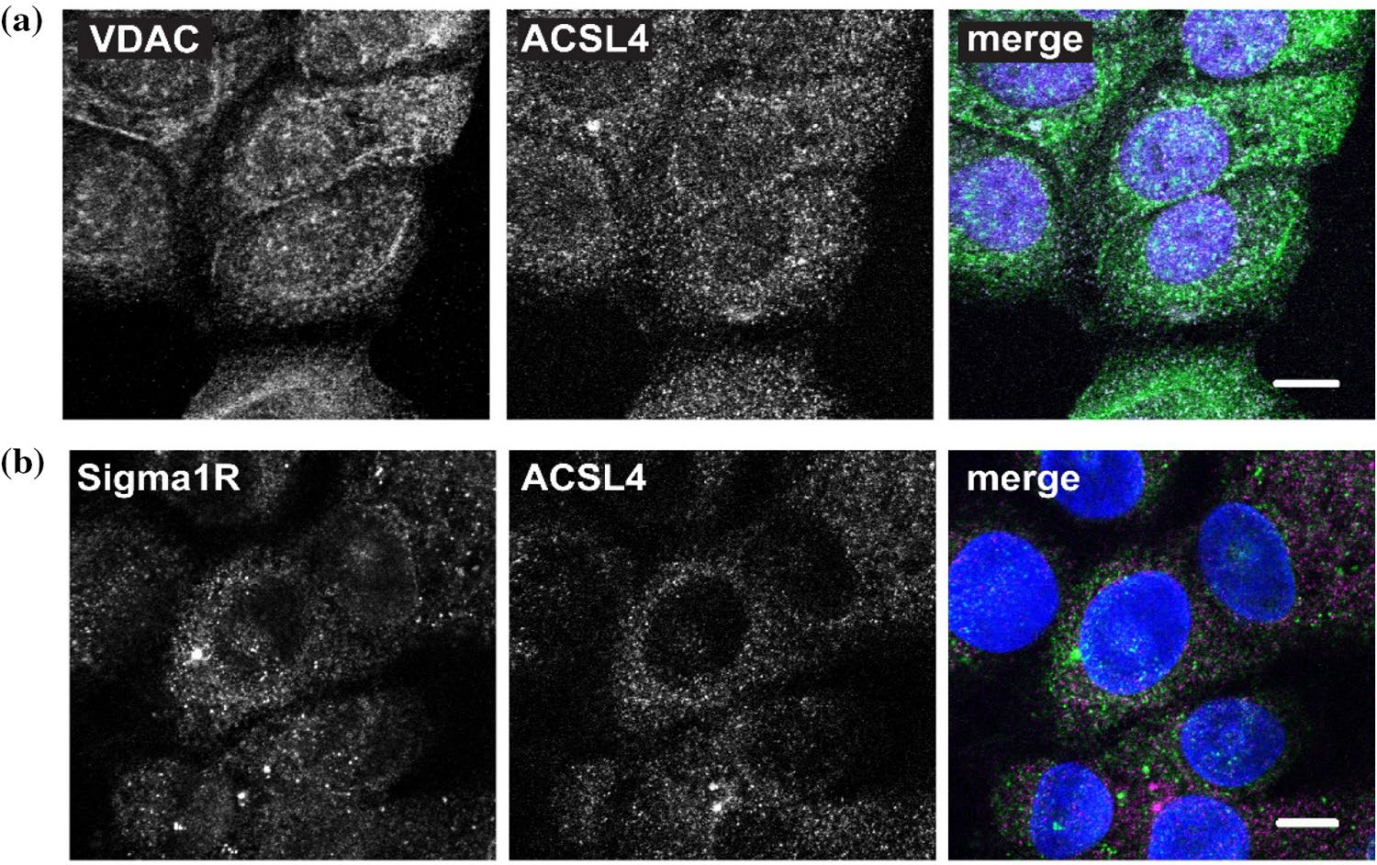
Imaging of the subcellular localisation of ACSL4 in MCF-7 cells. MCF-7 cells were formalin fixed and stained with antibodies to **a** VDAC (green) or **b** Sigma1R (green). ACSL4 (magenta) and chromatin counterstained with Hoechst (blue). Samples were imaged using confocal microscopy. Scale bars, 10 µm. (Color figure online)

To gain further insights into the intracellular targeting of ACSL4, we used confocal laser-scanning immunofluorescence microscopy and co-immunostained for ACSL4 in combination with the mitochondrial protein VDAC or the putative MAM marker Sigma1R (Fig. 7). We observed that ACSL4 had a cytoplasmic pattern of staining consistent with an endoplasmic reticulum localisation, and furthermore, there was no overlap between ACSL4 and VDAC. These results allowed us to rule out a mitochondrial localisation for this enzyme. We found that Sigma1R was localised to several membrane structures including perinuclear vesicles and the centrosome but exhibited very little overlap with ACSL4. This was unexpected given previous reports that Sigma1R was a MAM-specific protein and indicates that this protein is localised to more than one organelle in MCF-7 cells.

**Fig. 8:**
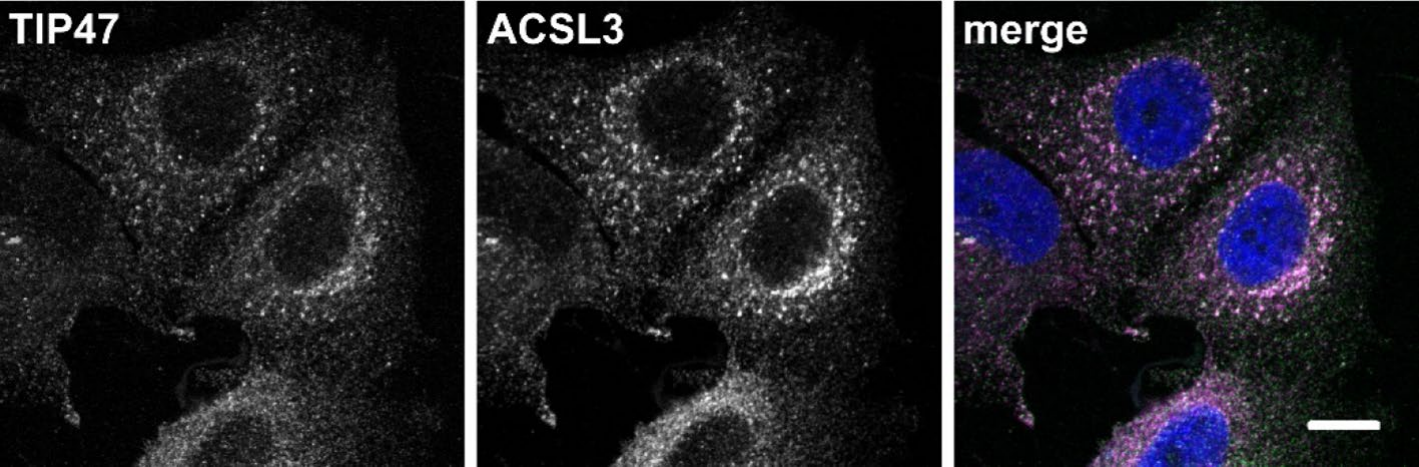
TIP47 and ACSL3 colocalise on membranes in the Golgi region. Cells were formalin fixed and stained with antibodies to TIP47 (green) and ACSL3 (magenta), before imaging using confocal microscopy. Cells were counterstained with Hoechst; scale bar, 10 µm. (Color figure online)

As with ACSL4, the steady-state localisation of ACSL3 was imaged in fixed cells by confocal laser-scanning indirect immunofluorescence experiments (Fig. 8). Using this technique, we observed that ACSL3 was present in vesicular puncta consistent with an endosomal localisation. Furthermore, in these experiments, we observed striking co-localisation between ACSL3 and TIP47. TIP47 is a protein originally identified as a mannose 6-phosphate receptor and Rab9-binding protein [39, 40] with endosomal trafficking functions. The substantial co-staining with ACSL3 noted here suggests that both proteins are targeted to the same trafficking pathway in MCF-7 cells. Interestingly, TIP47 is also known as PLIN3 and has been identified as a lipid droplet-associated protein in cells specialised for fat storage such as adipocytes [41]. However, our results indicate that in the cell lines investigated herein, TIP47 and ACSL3 are highly enriched on TGN–endosomal membranes.

These results shed new light on the intracellular organisation of fatty acid metabolism in cancer cells and demonstrate that at least in the HT1080 and MCF-7 cell lines, ACSL3 and ACSL4 have non-overlapping intracellular distributions. Furthermore, these results are in concordance with the concept that fatty acid activation is a highly compartmentalised process with fatty acids species of different acyl chain lengths and saturation being activated at specific subcellular sites. In the context of the present study, this could mean that the main ACSL3 substrates such as palmitate are preferentially activated at the TGN, while the ACSL4 substrate arachidonate would be activated once it is in contact with the extensive endoplasmic reticulum membrane. The high degree of overlap with calnexin indicates that the ACSL4 product arachidonyl-CoA could be made available for energy release via β-oxidation in mitochondria at MAM contact sites. However, as the endoplasmic reticulum is an important site for phospholipid synthesis it is also possible that the ACSL4 lipid product could be incorporated in the acyl chain components of glycerophospholipids such as phosphatidylinositol which are synthesised on ER membranes. However, the metabolic fate the acyl-CoA products potentially generated by ACSL3 on TGN/endosome membranes is less predictable. One possibility is that the ACSL3-derived acyl CoAs may be directly channelled to the generation of lipids required for vesicular trafficking processes such as the inositol phospholipids. We did note a high degree of gradient overlap between ACSL3 and PI4KIIα which may be suggestive of a link between ACSL3 and phospholipid metabolism on the TGN endosomal pathway, but further work is required to characterise the multiple metabolic steps and enzymes that would be required to establish a direct link between ACSL3 activity and inositol phospholipid acyl chain composition at this vesicular trafficking hub. However, the Golgi is a site for remodelling of acyl chain composition via the Land’s cycle, and we speculate that the ACSL3-activated lipid products may feed into this pathway via AGPAT3/LPAAT3 [42] and that this could potentially contribute to membrane tubulation and vesicle formation processes in this compartment. Finally, there is some evidence that the biological activity of ACSL3 is also determined by non-catalytic elements of the enzyme: recombinant, catalytically inactive ACSL3 can localise to lipid droplets [14]; non-catalytic ACSL3 can directly bind Lyn [15] and ACSL3 is also known to enhance the expression of lipogenic genes [43]. These observations in combination suggest that the biological functions of the ACSL enzymes cannot be completely understood solely in terms of their catalytic activities and that future work should aim to elucidate the functional importance of their distinct intracellular localisations.

In terms of furthering our understanding of the roles of ACSL enzymes in cancer, our results demonstrate that the expression patterns of these enzymes differ significantly in sarcoma samples and also that they are differentially localised in cancer cells. Although further work is necessary to understand the ramifications of these findings, they are nevertheless consistent with separable isoform-specific biological and pathological functions for the ACSLs and this may be useful information when considering therapeutic strategies to specifically target either enzyme in malignant disease.

## Electronic supplementary material

Below is the link to the electronic supplementary material.

Supplementary material 1 (XLSX 15 KB)

Supplementary material 2 (CSV 2 KB)

Supplementary material 3 (CSV 2 KB)

## Abbreviations

ACSL: Long chain fatty acyl CoA synthetase
EGFR: Epidermal growth factor receptor
HMGCR: HMG CoA reductase
HRP: Horse radish peroxidase
MAM: Mitochondrial-associated membrane
PI4KIIα: Phosphatidylinositol 4-kinase type IIα
PNS: Post-nuclear supernatant
SDS: Sodium-dodecyl-sulphate
TGN: *Trans*-Golgi network
